# First Report on the Rapid Detection and Identification of Methicillin-Resistant *Staphylococcus aureus* (MRSA) in Viable but Non-culturable (VBNC) Under Food Storage Conditions

**DOI:** 10.3389/fmicb.2020.615875

**Published:** 2021-01-07

**Authors:** Aifen Ou, Kan Wang, Yanxiong Mao, Lei Yuan, Yanrui Ye, Ling Chen, Yimin Zou, Tengyi Huang

**Affiliations:** ^1^Department of Food, Guangzhou City Polytechnic, Guangzhou, China; ^2^Center for Translational Medicine, The Second Affiliated Hospital of Shantou University Medical College, Shantou, China; ^3^Key Laboratory of Respiratory Disease of Zhejiang Province, Department of Respiratory and Critical Care Medicine, Second Affiliated Hospital of Zhejiang University School of Medicine, Hangzhou, China; ^4^College of Food Science and Engineering, Yangzhou University, Yangzhou, China; ^5^School of Biological Science and Engineering, South China University of Technology, Guangzhou, China; ^6^School of Food Science and Engineering, Guangdong Province Key Laboratory for Green Processing of Natural Products and Product Safety, South China University of Technology, Guangzhou, China; ^7^Department of Laboratory Medicine, The Second Affiliated Hospital of Shantou University Medical College, Shantou, China

**Keywords:** VBNC (viable but non-culturable), MRSA – methicillin-resistant *Staphylococcus aureus*, food storage, *mecA*, *femA*

## Abstract

Formation of viable but non-culturable (VBNC) status in methicillin-resistant *Staphylococcus aureus* (MRSA) has never been reported, and it poses a significant concern for food safety. Thus, this study aimed to firstly develop a rapid, cost-effective, and efficient testing method to detect and differentiate MRSA strains in the VBNC state and further apply this in real food samples. Two targets were selected for detection of MRSA and toxin, and rapid isothermal amplification detection assays were developed based on cross-priming amplification methodology. VBNC formation was performed for MRSA strain in both pure culture and in artificially contaminated samples, then propidium monoazide (PMA) treatment was further conducted. Development, optimization, and evaluation of PMA-crossing priming amplification (CPA) were further performed on detection of MRSA in the VBNC state. Finally, application of PMA-CPA was further applied for detection on MRSA in the VBNC state in contaminated food samples. As concluded in this study, formation of the VBNC state in MRSA strains has been verified, then two PMA-CPA assays have been developed and applied to detect MRSA in the VBNC state from pure culture and food samples.

## Introduction

Food safety has been found to be a leading concern for public health worldwide, and food pathogens remain the major factor as a causative for foodborne diseases (Deng et al., 2015a). A few microorganisms are top food pathogens, among which methicillin-resistant *Staphylococcus aureus* (MRSA) is an important one (Monteiro et al., 1997; Corrente et al., 2007). Since the first report in 1961, MRSA has become one of the leading human pathogens (Salisbury et al., 1997; Xu et al., 2010a; Wang et al., 2012). Firstly considered to be a major nosocomial pathogen, MRSA has been found to be an important foodborne pathogen in recent years. Aside from its biofilm formation capability and thus known to be a typical biofilm former, MRSA could also produce different types of toxins and is responsible for various human diseases (Kadariya et al., 2014; Miao et al., 2017a,b). In recent years, a number of foodborne outbreaks were reported to be caused by MRSA (Voss et al., 2005; Xu et al., 2017, 2019).

Prior to consumption, the most effective way to prevent food contamination by MRSA is accurate and rapid detection (Fang et al., 2009; Zhao et al., 2010b,c; Xu et al., 2020a). Currently, the gold standard for identification of MRSA is culturing and colony-forming unit (CFU) counting (Felten et al., 2002; Deng et al., 2015b). Routine detection will provide results on the random selected food samples as an indicator for the microorganisms inside such samples. However, such methodologies are based on the culturing, and microorganisms are capable of formation of the viable but non-culturable (VBNC) state. When entering the VBNC state, microorganisms are no longer culturable and thus yield false-negative results by culturing (Xu et al., 2011b; Liu et al., 2018a,b,d). Not limited within food safety, culturing is also the routine detection method for clinical strains (Wang et al., 2014, 2018; Xie et al., 2017a,b). Therefore, formation of the VBNC state is an important issue for both food safety and clinicians.

Concerning the VBNC formation and identification, rapid detection of the VBNC state of MRSA is of urgent necessity and importance, along with the formation of the VBNC state (Liu et al., 2016c, 2017a,b,c). In this study, formation of the VBNC state has been performed on MRSA strains, then development and evaluation of an isothermal nucleic acid amplification-based propidium monoazide (PMA) detection assay have been conducted on MRSA.

## Materials and Methods

### Bacterial Strains and Culturing

A total of five MRSA strains and 18 non-MRSA strains, including *Escherichia coli*, *Salmonella*, *Listeria monocytogenes*, *Vibrio parahaemolyticus*, *and Pseudomonas aeruginosa*, are included in this study. All strains had been previously identified by PCR and sequencing on the staphylococci species-specific target *femA*, methicillin-resistant gene *mecA*.

### Study Design

Two targets were selected to differentiate MRSA and non-MRSA strains, and one more target was based on an important toxin factor. In detail, the protocol was designed to (i) distinguish between *S. aureus* and coagulase-negative staphylococci or non-*S. aureus* strains based on amplification of the *S. aureus*-specific *femA* gene and (ii) distinguish methicillin-resistant *Staphylococcus* and methicillin-susceptible *Staphylococcus* based on amplification of the *mecA* gene. The sequences are as follows: *femA*: 4s-TCAAA TCGCGGTCCAGTG; 5a-AACCAATCATTACCAGCA; 2a/1s-T ACCTGTAATCT CGCCAT AACATCGTTGTCTATACCT; 2a-TACCTGTAATCTCGCCAT; 3a-GGTAAATATGGATCGATA TG. *mecA*: 4s-GCGATAATGGTGAAGTAG; 5a-GATCAATGT TACCGTAGTT; 2a/1s-TTACGATCCTGAATGTTT ATGACT GAA CGTCCGATA; 2a-TTACGATCCTGAATGTTT; 3a-TCTT TAACGCCTAAACTA.

### Strains Processing and Template DNA Preparation

Development and evaluation of CPA assay were performed on a total of five MRSA strains and 18 non-MRSA strains, of which *S. aureus* strains are isolated from raw milk and pork. All strains used in this study had been preliminarily identified. Crude DNA from MRSA and other bacteria strains used as template for amplification was prepared from overnight trypticase soy broth (TSB, Huankai Microbial, Guangzhou, China) culture, and DNA was extracted using a DNA Extraction Kit (Dongsheng Biotech, Guangzhou, China) according to the manufacturer’s instructions. The concentration and quality of the extracted DNA were measured using a Nano Drop 2000 (Thermo Fisher Scientific Inc., Waltham, MA, United States) at 260 and 280 nm. The processed DNA was stored at −20°C until further use.

### Development, Optimization, and Evaluation of CPA Assays

**FIGURE 1:**
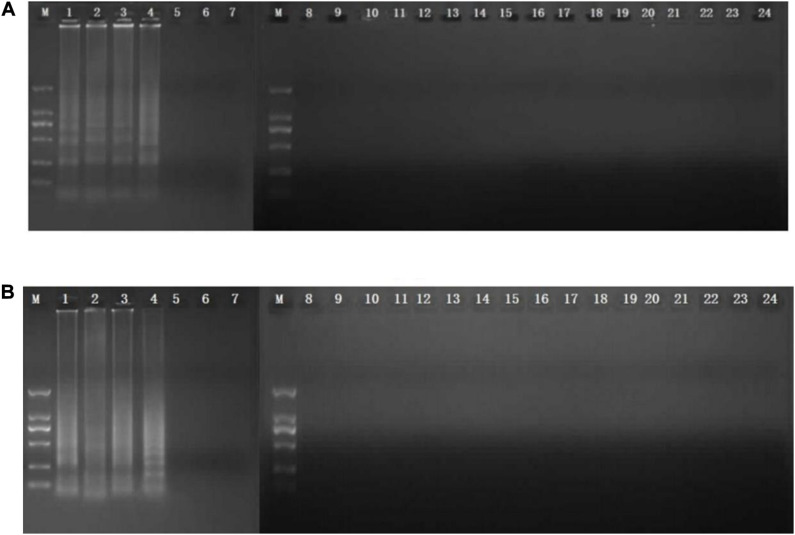
Specificity of CPA detection for different strains by 1.5% agarose gel electrophoresis and mixed chromogenic agent. For *femA* **(A)** and *mecA* **(B)** genes, lanes/tubes 1–5, *Staphylococcus aureus* 971311004, 0313113664, 0314030668, and 10071; lanes/tubes 5–24; non-MRSA strains, negative control.

**FIGURE 2:**
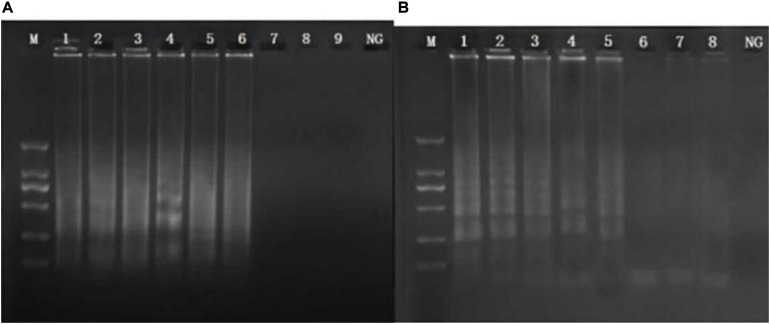
Sensitivity of the CPA assay in genomic DNA by 1.5% agarose gel electrophoresis. Sensitivity from 10,071 of *femA* **(A)** and *mecA* **(B)** genes. M, DNA marker; lanes 1–8, 3.0 ng/μl, 300 pg/μl, 30 pg/μl, 3 pg/μl, 300 fg/μl, 30 fg/μl, 3 fg/μl, and 300 ag/μl; NG, negative control.

CPA reaction was carried out in a total of 26-μl reaction mixture containing 20 mM Tris–HCl, 10 mM (NH_4_)_2_SO_4_, 10 mM KCl, 8 mM MgSO_4_, 0.1% Tween 20, 0.7 M betaine (Sigma, United States), 1.4 mM of dNTP mix, 8 U of Bst DNA polymerase large fragment (NEB, United States), 1.0 μM primer of 2a/1s, 0.5 μM (each) primer of 2a and 3a, 0.6 μM (each) primer of 4s and 5a, 1 μl DNA template, and 1 μl mixed chromogenic agent, and the total reaction mixture was made up to 26 μl with nuclease-free water (Xu G. et al., 2012; Zhang et al., 2015). The mixed chromogenic agent consists of 0.13 mM calcein and 15.6 mM MnCl_2_⋅4H_2_O. CPA reaction system was carried out at 63°C for 1 h and then heated at 80°C for 2 min to stop the amplification reaction. The negative control was performed by the 1 μl nuclease-free water instead of DNA template. PCR reaction was performed in a total 25 μl reaction system with five primers (4s, 5a, 2a, 1s, 2a, and 3a). The protocol of PCR was 95°C for 5 min, followed by 32 cycles of amplification at 95°C for 30 s, 52°C for 30 s, 72°C for 35 s, and final amplification at 72°C for 5 min. The specificity of primers was evaluated by amplification of the genomic DNA extracted from five MRSA strains isolated from raw milk and pork and 18 non-target bacteria, including *E. coli*, *Salmonella*, *L. monocytogenes*, *V. parahaemolyticus*, and *P. aeruginosa.* To study the sensitivity reaction on the DNA solution, 10-fold serial dilutions of total genomic DNA were subjected to CPA in triplicate. The products were detected by 1.5% agarose gel electrophoresis. The CPA assays for *femA* and *mecA* were established using MRSA 10071. The amplified products were detected by 1.5% agarose gel electrophoresis, and the bands were observed under UV light. In addition, 1 μl mixture chromogenic agent (MgCl_2_ and calcein) were added into the reaction system, wherein the dye color simultaneously changed from original to green in the positive sample or water retained the original orange color. The CPA assay was evaluated for its specificity using five MRSA strains and non-MRSA strains as control. Of these strains, only MRSA strains were amplified. No cross-reaction was found with all the related non-MRSA microorganism strains, indicating the high specificity of the designed primers (Figures 1, 2).

### Artificial Contamination of Food Samples

In this study, Cantonese pastry has been selected to be the food sample for artificial contamination and further detection. Rice and flour products are the major food type in China, which takes up the largest consumption market. Cantonese pastry is one of the most common for rice and flour products in China and thus was selected. For artificial contamination, 25 g of frozen Cantonese pastry (Guangzhou Restaurant, Guangzhou, China) was added to 225 ml of 0.9% NaCl, which was sterilized as food samples and contaminated by MRSA strains. The strains were incubated overnight (∼10^8^ CFU/ml) in TSB (Huankai Microbial, Guangzhou, China), which were mixed with the food samples to the final concentration of 10∼10^8^ CFU/ml. The contaminated food samples were subjected to complex preprocessing, then extracted with a rapid DNA processing, as described previously.

### Formation of Viable but Non-culturable State and Application of PMA-CPA

The developed CPA assays were utilized to detect the VBNC cells. The experimental strains were cultured to the exponential growth period. To induce the VBNC state of MRSA, the culture was diluted to the final concentration at 10^8^ CFU/ml with food homogenate (Cantonese pastry, steamed bread, rice flour; Guangzhou Restaurant, Guangzhou, China). Then, they were stored at −20°C to induce the VBNC state for further use of PMA-CPA. The trend of the number of culturable bacteria was used to make sure that the cells enter into culturable state. And culturable and viable cell enumerations were preformed every 3 days by traditional culture method. The LIVE/DEAD BacLight^TM^ kit (Thermo Fisher Scientific, United States) was performed under fluorescence microscope after the culturable colonies no longer form on agar medium. After confirmation of the VBNC state, PMA-CPA has been applied for detection.

## Results

### Development of CPA Assays

The analytical sensitivity of the CPA assay for MRSA was measured using 10-fold serial dilutions of MRSA. After CPA reaction, it revealed that the DNA detection limit of CPA were 30 fg/μl for *femA*, 300 fg/μl for *mecA* (Figures 1, 2). These results indicated that the analytical sensitivity and specificity of CPA are distinctly higher than those of conventional PCR.

### Application of the CPA Assays in Artificially Contaminated Food

**FIGURE 3:**
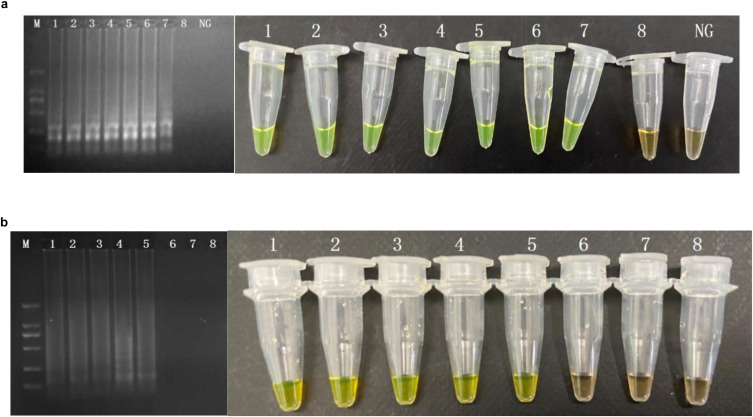
Sensitivity of the CPA by 1.5% agarose gel electrophoresis in food samples of *femA* **(a)**, *mecA* **(b)**. M, DNA marker; lanes 1–8, 10^7^ CFU/ml, 10^6^ CFU/mL, 10^5^ CFU/mL, 10^4^ CFU/mL, 10^3^ CFU/mL, 10^2^ CFU/mL, 10^1^ CFU/mL, 1 CFU/mL.; NG, negative control.

Sensitivity values of CPA and PCR assays for MRSA in food samples (rice, frozen pastry, and steamed bread) were 10^2^ CFU/ml and 10^4^ CFU/ml (Figure 3).

### Formation of Viable but Non-culturable and Evaluation of PMA-CPA Assay and in Food Samples

**FIGURE 4:**
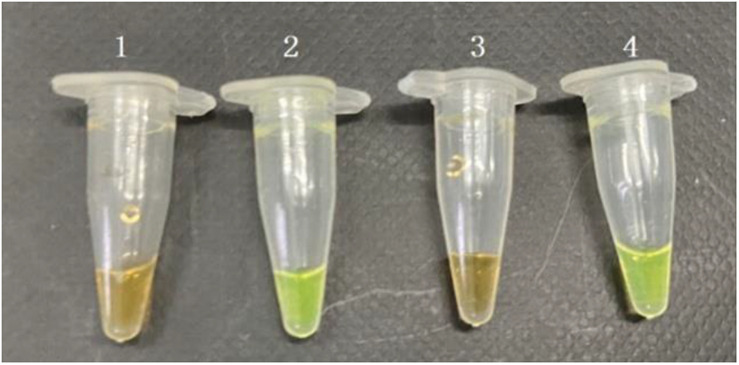
Detection of the viable but non-culturable (VBNC) state of *S. aureus* in pure cultures and food samples by PMA-CPA assays. 1, dead cells in pure cultures; 2, VBNC cells in pure cultures; 3, dead cells in food samples; 4, VBNC cells in food samples.

The VBNC status of MRSA has been studied according to a previous procedure. After confirmation of the VBNC status, the VBNC cell was added to pure culture and food samples. The PMA agent was used at the concentration of 5 μg/ml. Subsequently, the detection samples mixed with PMA were incubated in the dark at room temperature for 10 min before the tubes were placed horizontally on ice exposed to a halogen lamp (650 W) at a distance of 15 cm for 15 min to complete the combination of DNA and PMA. The mixed samples were centrifuged at 10,000 rpm for 5 min, and the precipitation under the tubes was processed by the rapid DNA processing methodology, which were prepared as DNA samples for PMA-CPA (Figure 4). The results showed that the VBNC cells in pure culture and food samples both can be detected by PMA-CPA assays.

## Discussion

Methicillin-resistant Staphylococcus aureus is considered one of the leading causes of food poisoning worldwide (Weese et al., 2010). A rapid and accurate detection method is required to detect MRSA as a result of its prevalence in various food samples (Crago et al., 2012). In this study, new approaches were evaluated for accurate detection of MRSA from food samples (Xu et al., 2009, 2020b, Xu Z. et al., 2012). CPA assays were developed utilizing a visual method. As a novel nucleic acid amplification method, CPA assays are both successfully performed to detect MRSA if there is co-occurrence of *S. aureus*-specific *femA* and methicillin-resistance marker *mecA* (Tacconelli et al., 2009; Miao et al., 2019). Since the invention of CPA technology, it has been employed for the detection of various bacteria. However, CPA is not suitable for the detection of multiple genes (*femA, mecA*) in the same system due to products that would differ in size (Xu et al., 2008, 2010b, 2011a; Yu et al., 2016; Liu et al., 2017d). Therefore, we established two CPA assays targeting *mecA* and *femA* to replace a single or multiple PCR assay.

Current methods available for detection of MRSA, including routine standard procedures (colony morphology, Gram staining, and testing of catalase, hyaluronidase, and coagulase), the Vitek 2 automated system, the API-Staph kit, immunological assays, mass spectrometry, and PCR (regular PCR as well as quantitative PCR), are time-consuming and required highly trained personnel (Graveland et al., 2011; Zhao et al., 2011; Liu et al., 2016a,b). This stricture can be overcome as the results from CPA methods require only a conventional heating block or a water bath, and the result can be judged through observing color change by adding SYBR Green I, calcein, or hydroxy naphthol blue (HNB) into the reaction tube (Liu et al., 2019). Furthermore, the CPA reaction can be accomplished in 40 min through designing accelerated primer (date was not shown), which is much shorter than the reaction times of PCR (Yulong et al., 2010; Zhao et al., 2010a). Overall, the detection of MRSA by CPA has resolved the major limitation of time consumption or complex procedure by culture-based methods and costly PCR molecular techniques (Liu et al., 2018c).

Taking specificity and sensitivity into consideration, CPA assays were developed as sensitive and rapid MRSA detection systems, and the experimental data indicated the utility of the established system in point-of care testing (Moellering, 2012; Xu et al., 2020a). The high specificity of CPA was evidenced by the successful detection of all MRSA, but not of non-target pathogens.

## Data Availability Statement

The raw data supporting the conclusions of this article will be made available by the authors, without undue reservation.

## Author Contributions

AO and KW conceived the study and participated in its design and coordination. YM, LY, LC, and YY performed the experimental work and collected the data. YZ and TH analyzed the data and wrote the manuscript. All authors read and approved the final manuscript.

## Conflict of Interest

The authors declare that the research was conducted in the absence of any commercial or financial relationships that could be construed as a potential conflict of interest.
