# Corrigendum: First report on the rapid detection and identification of methicillin-resistant *Staphylococcus aureus* (MRSA) in viable but non-culturable (VBNC) under food storage conditions

**DOI:** 10.3389/fmicb.2022.1006715

**Published:** 2022-08-22

**Authors:** Aifen Ou, Kan Wang, Yanxiong Mao, Lei Yuan, Yanrui Ye, Ling Chen, Yimin Zou, Tengyi Huang

**Affiliations:** ^1^Department of Food, Guangzhou City Polytechnic, Guangzhou, China; ^2^Center for Translational Medicine, The Second Affiliated Hospital of Shantou University Medical College, Shantou, China; ^3^Key Laboratory of Respiratory Disease of Zhejiang Province, Department of Respiratory and Critical Care Medicine, Second Affiliated Hospital of Zhejiang University School of Medicine, Hangzhou, China; ^4^College of Food Science and Engineering, Yangzhou University, Yangzhou, China; ^5^School of Biological Science and Engineering, South China University of Technology, Guangzhou, China; ^6^School of Food Science and Engineering, Guangdong Province Key Laboratory for Green Processing of Natural Products and Product Safety, South China University of Technology, Guangzhou, China; ^7^Department of Laboratory Medicine, The Second Affiliated Hospital of Shantou University Medical College, Shantou, China

**Keywords:** VBNC (viable but non-culturable), MRSA – methicillin-resistant *Staphylococcus aureus*, food storage, *mecA*, *femA*

In the published article, there was an error regarding the affiliation(s) for “Yimin Zou.” Instead of “Yimin Zou^1*^” it should be “Yimin Zou^3*^.”

The authors apologize for this error and state that this does not change the scientific conclusions of the article in any way. The original article has been updated.

## Publisher's note

All claims expressed in this article are solely those of the authors and do not necessarily represent those of their affiliated organizations, or those of the publisher, the editors and the reviewers. Any product that may be evaluated in this article, or claim that may be made by its manufacturer, is not guaranteed or endorsed by the publisher.

